# Adaptive genetic variation underlies biocomplexity of Atlantic Cod in the Gulf of Maine and on Georges Bank

**DOI:** 10.1371/journal.pone.0216992

**Published:** 2019-05-24

**Authors:** G. V. Clucas, L. A. Kerr, S. X. Cadrin, D. R. Zemeckis, G. D. Sherwood, D. Goethel, Z. Whitener, A.I. Kovach

**Affiliations:** 1 Department of Natural Resources, University of New Hampshire, Durham, NH, United States of America; 2 Gulf of Maine Research Institute, Portland, ME, United States of America; 3 School for Marine Science & Technology, University of Massachusetts Dartmouth, New Bedford, MA, United States of America; 4 Department of Agriculture and Natural Resources, Rutgers University, Toms River, NJ, United States of America; 5 F/V Ellen Diane, Hampton, NH, United States of America; University of Iceland, ICELAND

## Abstract

Atlantic cod (*Gadus morhua*) populations in the Gulf of Maine (GoM) are at a fraction of their historical abundance, creating economic hardships for fishermen and putting at risk the genetic diversity of the remaining populations. An understanding of the biocomplexity among GoM populations will allow for adaptive genetic diversity to be conserved to maximize the evolutionary potential and resilience of the fishery in a rapidly changing environment. We used restriction-site-associated DNA sequencing (RADseq) to characterize the population structure and adaptive genetic diversity of five spawning aggregations from the western GoM and Georges Bank. We also analyzed cod caught in the eastern GoM, an under-sampled area where spawning aggregations have been extirpated. Using 3,128 single nucleotide polymorphisms (SNPs), we confirmed the existence of three genetically separable spawning groups: (1) winter spawning cod from the western GoM, (2) spring spawning cod, also from the western GoM, and (3) Georges Bank cod. Non-spawning cod from the eastern GoM could not be decisively linked to either of the three spawning groups and may represent a unique component of the resource, a mixed sample, or cod from other unsampled source populations. The genetic differentiation among the three major spawning groups was primarily driven by loci putatively under selection, particularly loci in regions known to contain genomic inversions on linkage groups (LG) 7 and 12. These LGs have been found to be linked to thermal regime in cod across the Atlantic, and so it is possible that variation in timing of spawning in western GoM cod has resulted in temperature-driven adaptive divergence. This complex population structure and adaptive genetic differentiation could be crucial to ensuring the long-term productivity and resilience of the cod fishery, and so it should be considered in future management plans.

## Introduction

Identifying stock boundaries of marine populations remains a challenge despite rapid advances in genetic stock identification methods due to the considerable complexity in the population structure of many marine species [1]. Most marine fishery resources are characterized by large population sizes and high dispersal ability, resulting in subtle patterns of population differentiation [2,3], the characterization and interpretation of which is complex [4]. Given advances in sequencing technologies, thousands to millions of genetic markers can now be used to study population structure, enabling high-resolution interpretations.

Gene flow is often used to infer demographic connectivity, so that groups connected by high gene flow are often considered as one continuous stock. However, populations can be demographically independent despite high gene flow [4]. Further, populations connected by high gene flow may lack genetic differentiation at neutral genomic markers yet exhibit differentiation at adaptive loci as a result of divergent selection pressures. Patterns of gene flow and adaptive genetic diversity contribute to biocomplexity among stocks in life history, morphology, and local adaptation that may be highly relevant to fisheries management [5–7]. Accordingly, there may be unique ecological and functional diversity among stocks [8–11] the maintenance of which could be key to ensuring adaptive capacity or evolutionary potential [12,13]. Biocomplexity may also confer resilience; loss of intra-specific genetic diversity has been linked to reduced population stability and reduced resilience to exploitation and changing environmental conditions [5,11,14–16]. The U.S. Endangered Species Act mandates that populations divergent at putatively adaptive loci warrant designation as separate management units even if they appear panmictic at neutral loci. Therefore, stock identification for fisheries management should aim to use thousands of genetic loci to aid in assessing biocomplexity, including both neutral and adaptive genetic diversity, among populations, especially in regions experiencing rapid environmental change.

**Fig 1:**
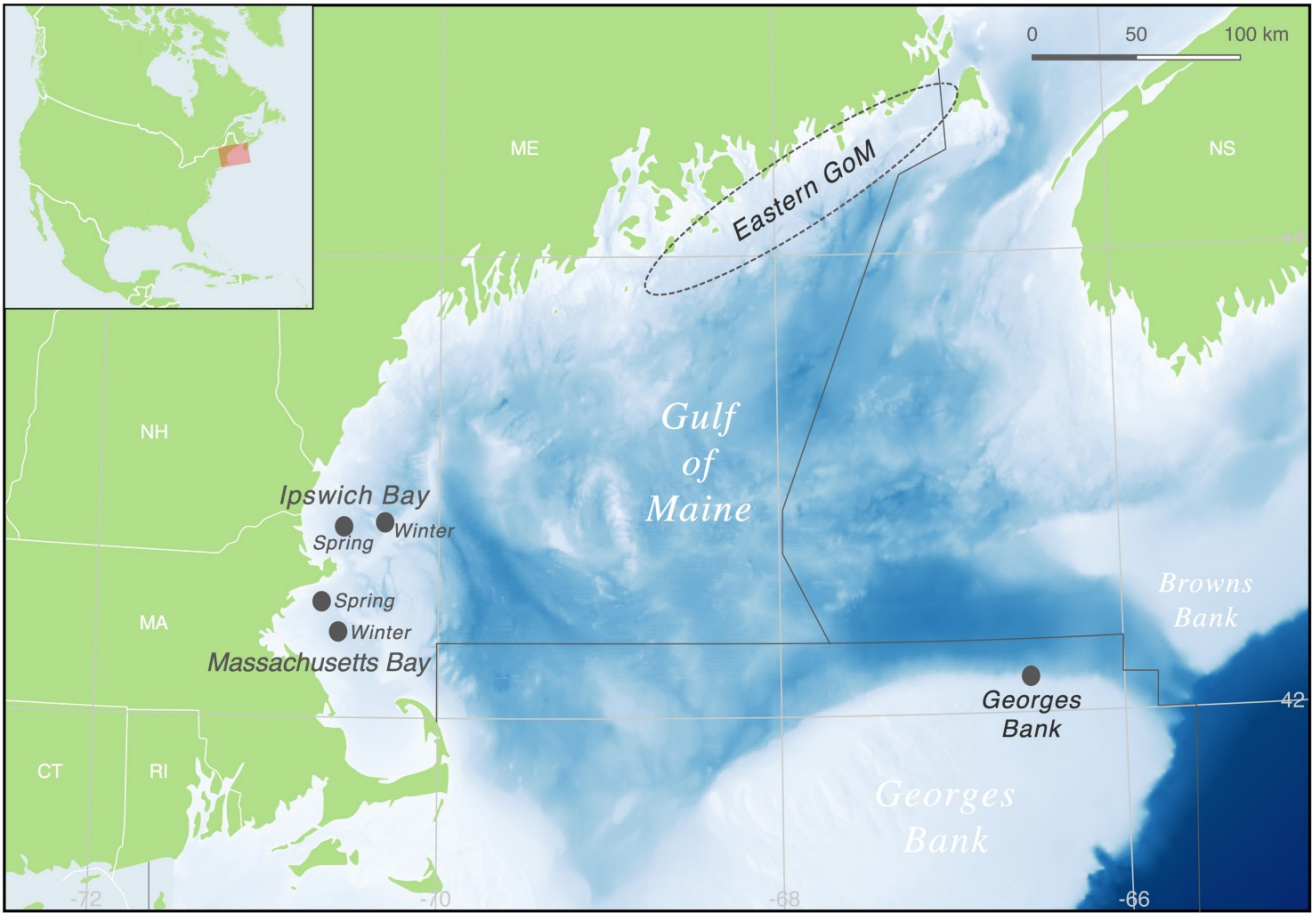
Locations of Atlantic cod groups analyzed in this study. Samples from Ipswich and Massachusetts Bays and Georges Bank were from known spawning grounds (dark gray circles), whereas the sentinel fishery targeted non-spawning cod in the eastern GoM (circled region). We do not have exact catch locations for all of the eastern GoM samples, as fishermen were not required to declare them, and so we show the overall area in which they were caught. The management unit boundaries of the GoM, Georges Bank, and Scotian Shelf are indicated by the thin black lines.

Population structure of Atlantic cod (*Gadus morhua*) is complex [1,17] and many investigations of the various populations and ecotypes that co-exist across the North Atlantic have made cod a canonical example of biocomplexity in a marine species. In U.S. waters, there is considerable biocomplexity in cod stocks [18–22] that is not reflected in the current stock assessment and management structure in place since 1972. The two management units consist of: 1) cod from Georges Bank, offshore of Cape Cod, and in the waters of southern New England; and 2) a group consisting of cod from the western, central, and eastern GoM (Fig 1). However, genetic data [23–28], differences in growth rates [29,30], timing of spawning [19,31], larval dispersal patterns [32,33], movement patterns [18,34–36], spawning site fidelity [37–39], and differences in life history strategy [40,41] all confirm that there is biocomplexity in US cod that conflicts with the stock designations (for a review see REF 20). The misalignment of stock designations with this biocomplexity is implicated as a contributing factor in the failure of these stocks to rebuild despite decades of intensive management [42].

Populations of Atlantic cod in the Gulf of Maine (GoM) are at a fraction of their historical abundance [22,43]. Currently, cod biomass is estimated at just 5–8% of its target level [43] and the fishery has been declared an economic disaster. Overexploitation has been implicated as one factor in the extirpation of historical spawning components in the GoM [19] and the low biomass of the remaining components puts the fishery at risk, especially as the GoM is warming faster than 99% of the global ocean [44–46]. Cod productivity appears to be closely linked to temperature [44,47,48] and adaptive diversity is likely going to be key for the resilience of the fishery in the face of this rapid ecosystem change.

In their investigation of genetic stock structure in the GoM using microsatellites, Kovach *et al*., [23] found genetic differentiation among spawning aggregations in the GoM, primarily stemming from outlier loci. This structure included differentiation among subpopulations that overlap spatially but differ in spawning season (i.e., genetically distinct winter- and spring-spawning populations). Barney *et al*., [24] also found that adaptive variation played a key role differentiating two spawning populations in the western GoM and one on Georges Bank, with differentiation in regions of the genome that were in high linkage disequilibrium (LD) on linkage groups (LG) 2, 7 and 12. These LD blocks are chromosomal inversions [49–51] and have been previously found to segregate between populations of cod in other regions spatially [52–55], temporally [6,56], between stationary and migratory ecotypes [50,51,54,55,57,58], and have been linked to spatial clines in temperature across the North Atlantic [52,53]. Therefore, despite potential gene flow among subpopulations within the GoM, there is growing evidence for adaptive differentiation among spawning aggregations [23,24].

To date, no genetic studies have focused on cod in the eastern GoM (from US waters off midcoast and eastern Maine). Ames’ [19] analysis of historical fishing grounds suggested that cod previously found in these areas comprised separate spawning components that were likely discrete from cod in the western GoM. Spawning components within the eastern GoM have been functionally extirpated [19] and since 2010, the Maine Center for Coastal Fisheries’ Sentinel Hook Survey Fishery has monitored cod abundance in the region to determine whether stocks are rebuilding [59,60]. Although spawning aggregations have not been observed recently, cod caught by the sentinel survey allow an opportunity to determine the level of connectivity between the eastern GoM and other regions. Characterizing the connectivity of these eastern GoM cod with the more robust western populations is vital for understanding the metapopulation structure of cod in the GoM and their potential for recovery [20].

In this study, we used genome-wide genetic data to analyze both the neutral and putatively adaptive population structure of cod in the GoM. We sampled seasonally divergent spawning aggregations in the western GoM to characterize gene flow and adaptive divergence between populations that spawn in the same locations but in different seasons. We also investigated connectivity among these western GoM spawning aggregations, a Georges Bank spawning aggregation, and cod caught by the sentinel fishery in the eastern GoM that were not in spawning condition. If there were distinct subpopulations in the eastern GoM, as hypothesized [19,20], and the cod caught in these areas today are remnants or resurgent of these subpopulations, then they could still harbor unique genetic signatures even if they were not spawning at the time of sampling. Alternatively, a lack of differentiation of eastern GoM fish may suggest that there has been contemporary movement of individuals into the eastern GoM, or that these fish were never in fact distinct from other regions.

## Methods

### Sample collection

For the western GoM, fin clip samples from spawning adult cod were collected on known spawning grounds in Ipswich and Massachusetts Bays at two distinct spawning seasons–late spring (May/June) and winter (December/January; Table 1). The National Marine Fisheries Service granted the GMRI permission to collect samples during fisheries closures for the sole purpose of scientific research via a Scientific Research Letter of Acknowledgment. The fishing vessel F/V Ellen Diane was not subject to the Magnuson-Stevens Act or fishery regulations at 50 CFR part 648 while collecting samples for scientific research. For Georges Bank, we used archived DNA samples from the study of Kovach *et al*., [23]. These samples were collected by a survey conducted by Canada’s Department of Fisheries and Oceans from spawning adults on the Northeast Peak in February 2006 and, although there is a temporal mismatch between these and the other samples used in this study (Table 1), previous work [23] and this study shows temporal stability in the population structure in the region. For the eastern GoM, fin clip samples were collected from non-spawning cod caught in the sentinel fishery survey from stations located throughout mid-coast and Downeast Maine, with a majority of the samples from the inshore and nearshore areas and few collected from offshore (Fig 1). Maturity stages were defined based on [61].

**Table 1:** Information about the samples analysed in this study.

Name	Sampling site	Collection date	Spawning conditions of individuals	N
Ipswich Bay spring	Ipswich Bay	May 2015	2R, 13U.	15
Ipswich Bay winter	Ipswich Bay	December 2014	2D, 5R, 16U, 1S	24
Massachusetts Bay spring	Massachusetts Bay	May & June 2013	3R, 18U, 3S	24
Massachusetts Bay winter	Massachusetts Bay	December 2013	2D, 14R, 5U, 3S	24
Georges Bank	NE Georges Bank	February 2006	6R, 12U	18
Eastern Gulf of Maine	Mid-coast to Downeast Maine	June, July & August 2014; June & August 2015	Unknown (non-spawning)	23

Spawning conditions: D = developing, R = ripe, U = ripe and running, S = spent.

N = number of individuals included in final dataset.

### Library preparation and sequencing

DNA was extracted from fin clips with a Qiagen DNeasy Tissue Kit (Qiagen, Valencia, CA, USA), following the manufacturer’s protocols. RAD libraries were prepared and sequenced by Floragenex Inc. (Eugene, OR, USA), following standard protocols of [62]. Libraries were prepared from 50 μL of DNA per sample with concentrations of 10–20 ng/μl. Restriction digestion was performed with the *SbfI* enzyme and 300–500 base pair fragments were size selected. Individual DNA samples were barcoded with uniquely indexed nucleotides, to allow for the pooling of 95 multiplexed individuals per library. The resulting RAD libraries were sequenced on two lanes of an Illumina HiSeq using Illumina 1 x 100 bp chemistries.

### Bioinformatics

Raw read quality was assessed with FastQC [63] and reads were demultiplexed using the process_radtags function from the Stacks v1.35 pipeline [64,65]. Adapter trimming and quality filtering were performed with skewer [66] using the following settings: trim adapters found anywhere in a read (-m any), maximum allowed error rate = 0.1 (-r 0.1), trim 3’ end until minimum quality of 20 is reached (-q 20), trim reads with mean quality < 20 (-Q 20), minimum read length allowed after trimming = 40 (-l 40), filter highly degenerative reads (-n). After this filtering, 15 individuals with fewer than 800,000 reads remaining were discarded from further analysis. These individuals had significantly fewer reads than other individuals and initial single nucleotide polymorphism (SNP) calling trials resulted in more than 30% missing data for these individuals. Therefore, to retain the maximum number of loci, we discarded these individuals before performing our final SNP calling and SNP filtering.

SNP calling was performed using the ipyrad v0.7.21 pipeline [67] mapping to the cod reference genome (gadMor2; [68]). This pipeline uses bwa-mem [69] with default settings for alignment to the reference genome and discards reads that do not map uniquely. We set the minimum depth per locus to five reads and the maximum to 50,000 reads within individuals. We set this high maximum depth filter due to the high depth of sequencing that some of our samples received (Figure A in S1 Appendix). Additional maximum depth filters were applied downstream (see below) to remove potential paralogs. We specified that a locus had to be shared by at least 70% of individuals in each group to be included in the final dataset, while all other ipyrad settings were left as defaults, including the maximum heterozygosity filter of 0.5.

After SNP calling, SNPs were further filtered using vcftools v0.1.15 [70] so that the final dataset included only: biallelic SNPs; one SNP per RADtag; SNPs with a minor allele frequency ≥ 5%; SNPs with less than 30% missing data per sampling location; SNPs with mean depth less than two standard deviations above the mean to avoid paralogs; and SNPs that were in Hardy-Weinberg equilibrium in at least half of the groups at the *p* < 0.01 significance level. PGDSpider [71] was used to convert the data between formats for downstream analyses.

### Putatively neutral SNP selection

To identify a putatively neutral SNP dataset for determining gene flow among groups, we first thinned the data using vcftools such that SNPs were at least 10 kb apart to avoid tightly linked SNPs. We then used the F_*ST*_ outlier method implemented in BayeScan [72] to identify outlier loci. We set the prior-odds of neutrality to 10 to favor the detection of outliers and used a q-value cut-off of 0.05 (this means 5% of identified loci are expected to be false positives). Furthermore, we removed SNPs located within regions of the genome with putative inversions. These inverted regions have elevated linkage disequilibrium, including inter-chromosomal linkage disequilibrium [73], and in some cases show elevated F_*ST*_ among spawning aggregations within the GoM [24]. Therefore, we discarded SNPs within these regions as well as the F_*ST*_ outliers, to create our putatively neutral SNP dataset, hereafter “neutral dataset”. Further analyses were conducted with and without these outlier loci and are referred to as the full dataset and neutral dataset, respectively.

### Population structure

Pairwise F_*ST*_ values between groups were calculated for the neutral and full SNP datasets with the Hudson estimator as it has been shown to be insensitive to variation in sample sizes and was found to be unbiased when tested on SNP datasets [74,75]. To calculate the Hudson estimator, we used the R script of Di Gaetano *et al*., [76], correcting for an error on line 14, and modified the script to estimate bootstrapped confidence intervals with 1000 replicates, using the ‘boot’ package in R and the percentile method for the confidence interval.

Population structure was visualized with both datasets using a principal components analysis (PCA), performed using the *adegenet* v2.1.1 packages in R [77–79]. Before performing the PCA, allele frequencies were scaled and centered using the *scaleGen* function, and missing values were replaced by the mean allele frequency. PCA was performed with the *dudi*.*pca* function.

Discriminant Analysis of Principal Components (DAPC, REF 78) was also performed to assess genetic differentiation. The *find*.*clusters* algorithm suggested the most likely number of clusters within the data was equal to one (*K* = 1) for both datasets, suggesting population differentiation was subtle, and so we used the *a priori* clustering of individuals by group instead. The number of principal components (PCs) to retain was estimated by cross-validation using the *xvalDAPC* function with 1000 replicates [80].

To verify that variation in sequencing depth and missing data did not produce erroneous signals of population structure, we created a more stringent SNP dataset, subsetting the final SNP dataset for SNPs with less than 5% missing data across all sampling locations and repeated the DAPC analysis, as above. With this stringent SNP dataset, we also recoded missing data as 1 and genotypes as 0 and performed a PCA using the prcomp() function in R, scaling and centering the data, to check that patterns of missing data were not driving population structure.

*Structure* v2.3.4 [81] was also used to find clusters in the full and neutral SNP datasets and to estimate the proportion of ancestry individuals inherited from each cluster, effectively assigning individuals to clusters. The admixture model with correlated allele frequencies was used in all cases, as we expected to find a high level of admixture and gene flow among groups. An initial run was performed with *K* = 1, allowing lambda to vary, to estimate the value of lambda that was then set in all subsequent runs. *Structure* was run both with and without sampling locations as priors to differentiate between subtle and strong population differentiation, and values of *K* from one to six were tested. Each analysis was run for 150,000 generations, discarding the first 50,000 as burn-in, and repeated ten times from a random seed for each value of *K*. *Structure Harvester* web v0.6.94 [82] was used to compare replicates, calculate the most likely value of *K* according to the Evanno method [83], and prepare files for CLUMPP [84]. CLUMPP aligns the results from replicate runs of Structure to check for multimodality and calculates the average membership coefficients of individuals to each cluster, ready for visualization with DISTRUCT v1.1 [85]. To check for hierarchical structure, we removed groups that appeared differentiated and then repeated the Structure analysis on the remaining groups.

To investigate patterns of genetic differentiation throughout the genome, we created Manhattan plots using the R package *qqman* for locus-specific F_*ST*_ values from the full dataset for all pairwise comparisons between groups. Locus-specific F_*ST*_ was calculated using the Weir and Cockerham [86] estimator in vcftools. Spring and winter spawning individuals from Ipswich Bay and Massachusetts Bay were grouped together into “spring spawners” and “winter spawners” for these comparisons due to the genetic similarity between bays in the same season.

## Results

### Sequencing, SNP calling, and filtering

After discarding individuals with low read counts, we called SNPs in a total of 128 individuals across our six sampling locations (Table 1). The average number of reads per individual was 3.34 million (range: 0.87–12.18 million), with on average 84% of reads retained after quality control, adapter trimming, and mapping. Despite uneven sequencing depth among individuals and groups (Figure A in S1 Appendix), patterns of population structure did not correspond to these differences in sequencing depth or the lane in which individuals were sequenced, indicating that the patterns of population structure were robust. For example, spring spawners from Ipswich Bay, which received high coverage and were sequenced in lane 1, were genetically most closely related to spring spawners in Massachusetts Bay (see *Population structure* section), which had received much lower coverage and were sequenced in lane 2.

We initially identified 73,495 SNPs across all RADtags using Ipyrad. After SNP filtering, we retained 3,128 SNPs which had a mean sequencing depth of 77.5X when averaging across all individuals (range 20.8–113 X, Figure B in S1 Appendix). This comprised our full SNP dataset which included potentially adaptive loci (Table A in S1 Appendix) and loci with up to 30% missing data per sampling location. To further verify that variation in sequencing depth and missing data did not produce erroneous signals of population structure, we further subsetted this dataset to remove SNPs with more than 5% missing data, creating a stringent SNP dataset of 1,660 SNPs. To obtain the neutral dataset, we thinned our full SNP dataset to remove any SNPs that were < 10kb apart, removed an additional 47 SNPs that were identified as outliers (the details of these are available on figshare: https://doi.org/10.6084/m9.figshare.7683938.v1) and another 106 SNPs located in regions in high linkage disequilibrium on LG 2, 7, and 12. This resulted in a neutral dataset of 2,689 SNPs.

### Population structure

When we used the full dataset, which included the neutral SNPs plus the 106 SNPs in inverted regions on LG 2, 7 and 12, and 47 outlier SNPs identified by *BayeScan*, all pairwise F_*ST*_ values between sampling locations were small, but confidence intervals did not overlap zero (Table 2). Comparisons between the bays in the western GoM showed stronger differentiation by spawning season than geographic location. Georges Bank was more differentiated from the western GoM than the eastern GoM. Lastly, the eastern GoM showed similar levels of differentiation with the western GoM winter spawners as with Georges Bank, but higher differentiation with the western GoM spring spawners.

**Table 2:** Pairwise F_ST_ values among all sampling locations, estimated using the Hudson estimator and the full SNP dataset. The weighted mean value is given above and the confidence interval is given below.

	Ipswich Bay spring	Massachusetts Bay spring	Ipswich Bay winter	Massachusetts Bay winter	Georges Bank
Massachusetts Bay spring	0.0073				
	0.0045–0.0108				
Ipswich Bay winter	0.0185	0.0166			
	0.0142–0.0233	0.0132–0.0199			
Massachusetts Bay winter	0.0173	0.0158	0.0085		
	0.0133–0.0221	0.0126–0.0194	0.0069–0.0103		
Georges Bank	0.0202	0.0190	0.0148	0.0157	
	0.0154–0.0255	0.0148–0.0241	0.0116–0.019	0.0125–0.0197	
Eastern GoM	0.0151	0.0171	0.0070	0.0101	0.0079
	0.0111–0.0192	0.0131–0.0220	0.0042–0.0106	0.0072–0.0139	0.0057–0.0100

**Fig 2:**
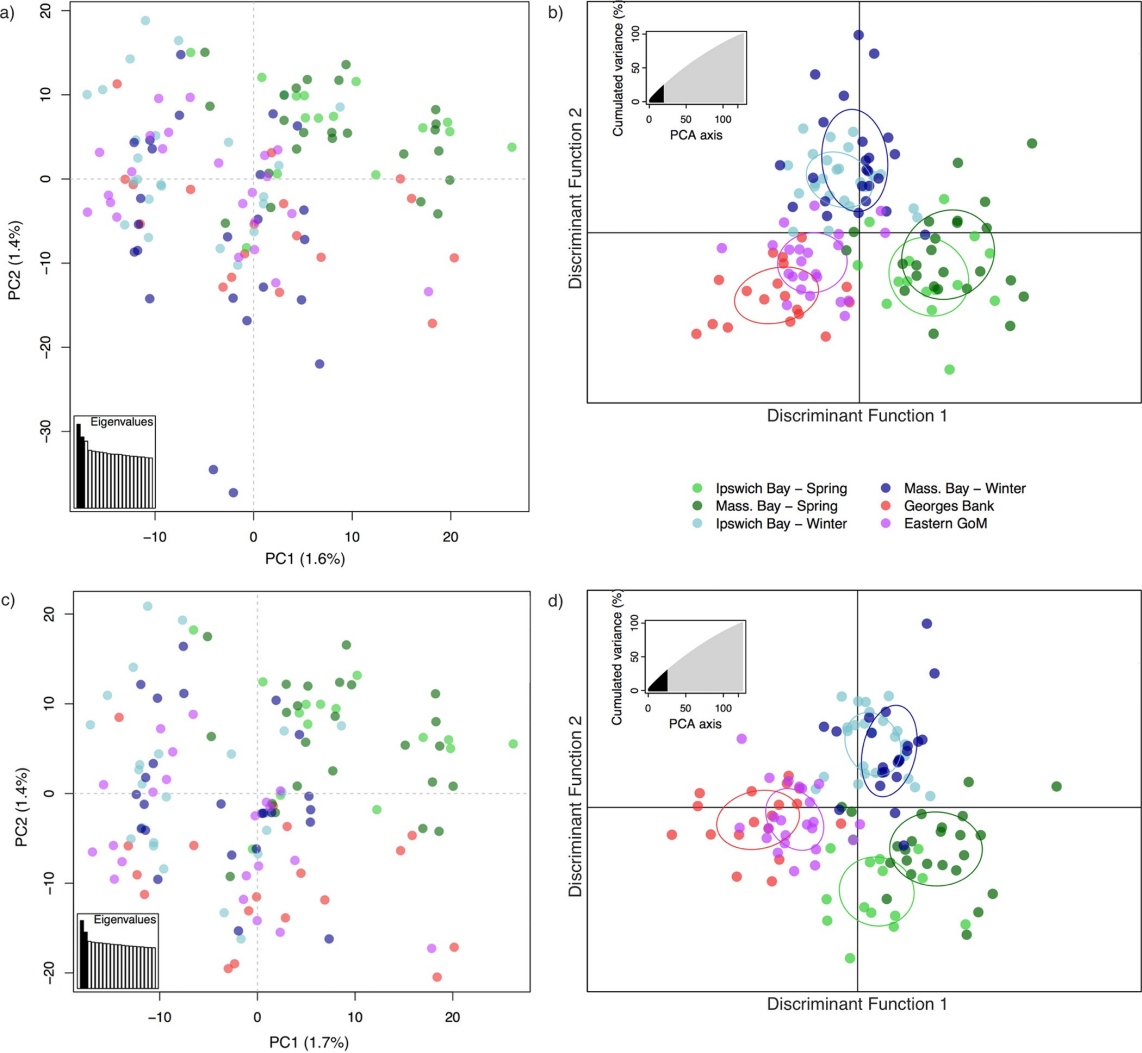
Population differentiation using the full SNP dataset that includes both neutral and outlier SNPs. a) PCA including all individuals, b) DAPC including all individuals, c) PCA after the Massachusetts Bay outlier individuals were removed, d) DAPC after the same Massachusetts Bay outlier individuals were removed. The percentage of the variation explained by each principle component is shown on the axis labels for the PCAs, and the cumulative percentage of variation explained by the discriminant functions is shown on the inset graphs for the DAPCs. The number of principle components retained in b) was 20 and in d) was 26, as assessed using cross-validation.

Results from PCA and DAPC showed patterns consistent with those described by the pair-wise F_*ST*_ comparisons and differentiation of the spring spawners. Individual scores formed three clusters on the PCA along PC1 (Fig 2A), which explained 1.6% of the variance. This three-cluster pattern is typical when there are two alternate forms of a large, high linkage disequilibrium genomic block driving genetic differentiation, because individuals can effectively be genotyped as having either of the homozygous states (clusters at the extremes of PC1) or the heterozygous state (the cluster in the middle of PC1). Spring spawners from Ipswich and Massachusetts Bays were found in the middle and right-hand cluster and shifted towards the upper right-hand corner of the plot; winter spawners were only found in the middle and left-hand cluster and were shifted towards the lower left-hand corner of the plot. Similar to the spring spawners, winter spawners from Ipswich and Massachusetts Bays overlapped with one another. Georges Bank and eastern GoM cod were found in all three clusters, but towards the lower half of the plot where the winter spawners were also found. There was no additional clustering in the PCA when other PCs were plotted. SNP loadings (Figure C in S1 Appendix) revealed that SNPs in the inverted region on LG 7 were largely responsible for the patterns along PC1, while SNPs in the inversion on LG 12 differentiated individuals along PC2. A single SNP on LG18 also seemed to have a large effect in differentiating populations on PC1. The clusters on the far left and right of PC1 correspond to individuals homozygous for the inverted and non-inverted regions of LG 7, while individuals in the middle cluster are heterozygous for the inversion. The differentiation along PC2 is the result of similar patterns stemming from the inversion on LG12.

DAPC provided an informative way to visualize these patterns of differentiation among the groups (Fig 2B). Spring spawners tended to group together into one cluster, winter spawners into a second cluster, and Georges Bank and eastern GoM into a third cluster when individuals were assigned to their *a priori* sampling group. The same clustering was also observed when the DAPC was repeated with the stringent SNP dataset of 1,660 loci (i.e. SNPs with only 5% missing data; Figure D in S1 Appendix) and there was no structure in the missing data (Figure E in S1 Appendix), confirming that the population structure we observed had a biological basis. The PCA plot using the full SNP dataset, and to a lesser degree the DAPC plot, revealed two individuals in the Massachusetts Bay winter spawning aggregation that appeared to be more genetically divergent than all others. We therefore removed these outliers and repeated both analyses (Fig 2C and 2D). The patterns we observed were stable after removal of these outlier individuals.

**Fig 3:**
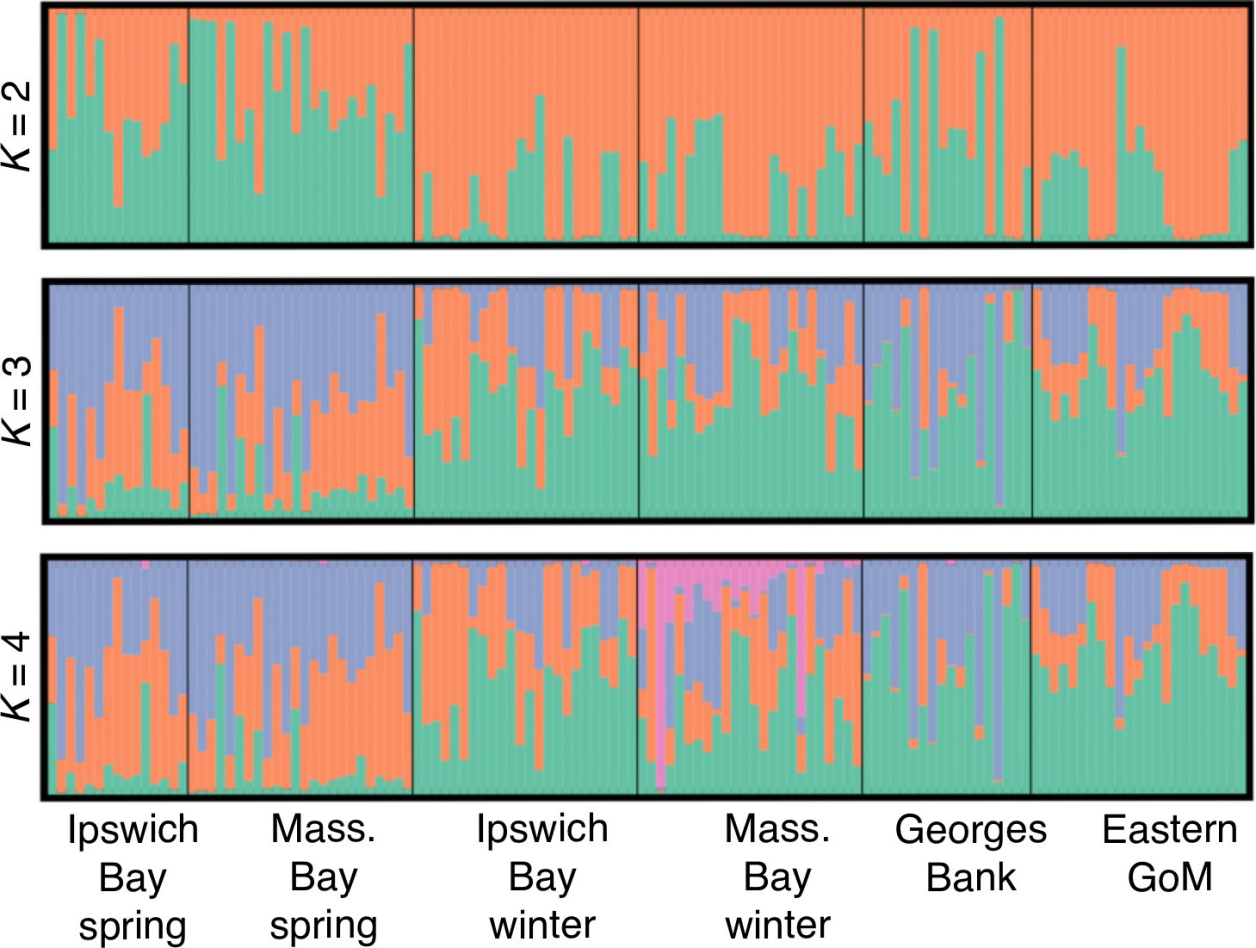
Individual assignment probabilities of sampled Atlantic cod to genetic clusters identified by Structure analysis. Bar plots show aggregated results for K = 2–4 from ten replicate runs using the full SNP dataset, which includes both neutral and outlier SNPs, with location priors. Geographic sampling locations are indicated below.

The differentiation of the spring spawners was also evident in a *Structure* analysis using the full SNP dataset. When *K* (number of populations) was varied from two to four, the spring spawners appeared differentiated from other groups (Fig 3). This pattern was evident in analyses both with location priors (Fig 3) and without location priors (Figure F in S1 Appendix). The Evanno method suggested *K* = 2 was the most likely value of *K* in both cases, although the estimated posterior probability of the data increased up to *K* = 4 (Figures F and G in S1 Appendix).

### Neutral population structure

When we analyzed the data after removing the 47 outlier SNPs and the 106 SNPs on LG 2,7, and 12 within the inversions, pairwise F_*ST*_ values among all groups were much smaller than for the full dataset. F_*ST*_ values were close to zero with the neutral dataset, although the confidence intervals again did not overlap zero, suggesting the possibility of some fine-scale differentiation among groups (Table 3). Pairwise F_*ST*_ was almost the same for two comparisons: Georges Bank *versus* eastern GoM and Ipswich Bay winter *versus* Massachusetts Bay winter (Table 3). The results could suggest that cod in these pairs of populations were under similar selection pressures to one another, since the inclusion of outlier SNPs did not change the levels of genetic differentiation between these pairs. Georges Bank and Massachusetts Bay winter spawners had on average slightly higher pairwise F_*ST*_ values compared to most other comparisons, suggesting subtle neutral differentiation of these two populations.

**Table 3:** Pairwise F_ST_ values estimated using the Hudson estimator and the neutral SNP dataset. The weighted mean value is given above and the confidence interval is given below.

	Ipswich Spring	Massachusetts Bay Spring	Ipswich Winter	Massachusetts Bay Winter	Georges Bank
Massachusetts Bay Spring	0.0047				
	0.0027–0.0067				
Ipswich Winter	0.0059	0.0068			
	0.0038–0.0080	0.0050–0.0088			
Massachusetts Bay Winter	0.0074	0.0097	0.0081		
	0.0053–0.0095	0.0077–0.0117	0.0062–0.0100		
Georges Bank	0.0082	0.0079	0.0090	0.0118	
	0.0056–0.0109	0.0058–0.0102	0.0067–0.0112	0.0097–0.0140	
Eastern GoM	0.0032	0.0052	0.0037	0.0070	0.0071
	0.0011–0.0052	0.0036–0.0067	0.0021–0.0054	0.0053–0.0087	0.0050–0.0093

**Fig 4:**
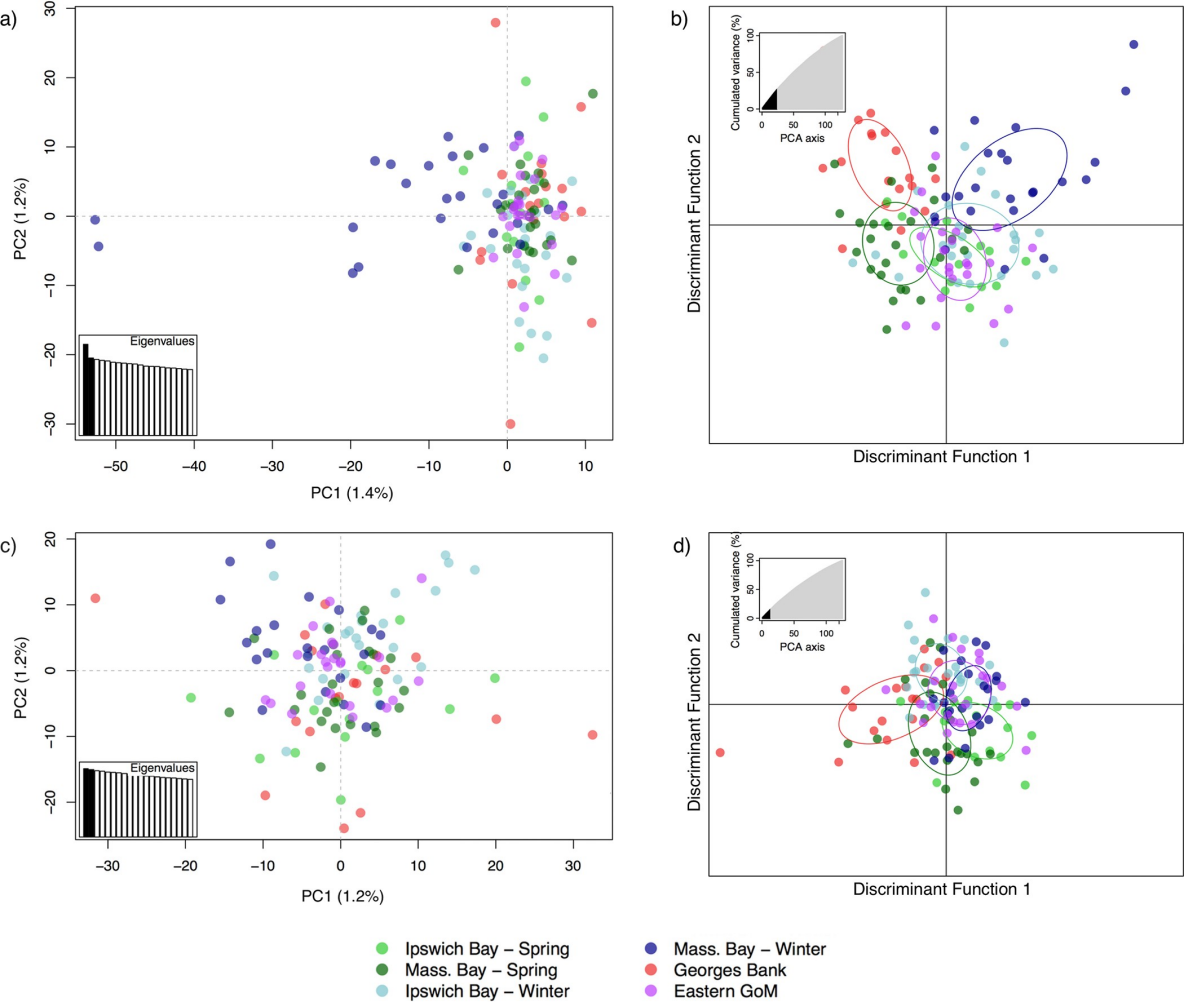
Population differentiation using the neutral dataset. a) PCA with all individuals; b) DAPC with all individuals. Removal of two outlying individuals from Massachusetts Bay winter largely collapses all groups in both c) PCA and d) DAPC. The percentage of the variation explained by each principle component is shown on the axis labels for the PCAs, and the cumulative percentage of variation explained by the discriminant functions is shown on the inset graphs for the DAPCs. The number of principle components retained in b) was 24 and in d) was 13, as assessed using cross-validation.

PCA and DAPC provided much less evidence for genetic differentiation among groups at putatively neutral SNPs in comparison to the analyses with the full dataset (Fig 4). PCA showed some limited patterns of neutral differentiation, with the Massachusetts Bay winter individuals clustering to the left and to a lesser extent the Ipswich Bay winter individuals clustering to the lower right portion of the plot (Fig 4A). DAPC suggested weak separation of Georges Bank, Massachusetts Bay spring, and Massachusetts Bay winter, with individuals from Ipswich Bay spring and winter and eastern GoM grouping together in the central portion of the plot (Fig 4B). Removal of the two outlier individuals from the Massachusetts Bay dataset caused most of the differentiation of Massachusetts Bay winter to disappear (Fig 4C and 4D) and caused the pattern in the DAPC to largely collapse, with a weak separation of Georges Bank and the two spring spawning populations remaining and broad overlap of the other three groups.

The lack of clear differentiation at neutral loci was also apparent with our *Structure* analyses (Figure H in S1 Appendix). The Evanno method for determining the ‘true’ value of *K* suggested *K* = 2 was most likely for analyses both with and without location priors. At values of *K* from two to four, the outlier individuals in Massachusetts Bay winter were assigned to a separate cluster, while other populations did not show clear patterns of differentiation (Figure H in S1 Appendix). This is not surprising since the neutral genetic differentiation among these populations is subtle and *Structure* is known to perform poorly when F_*ST*_ is less than 0.05 [87].

### Genome-wide patterns of differentiation

**Fig 5:**
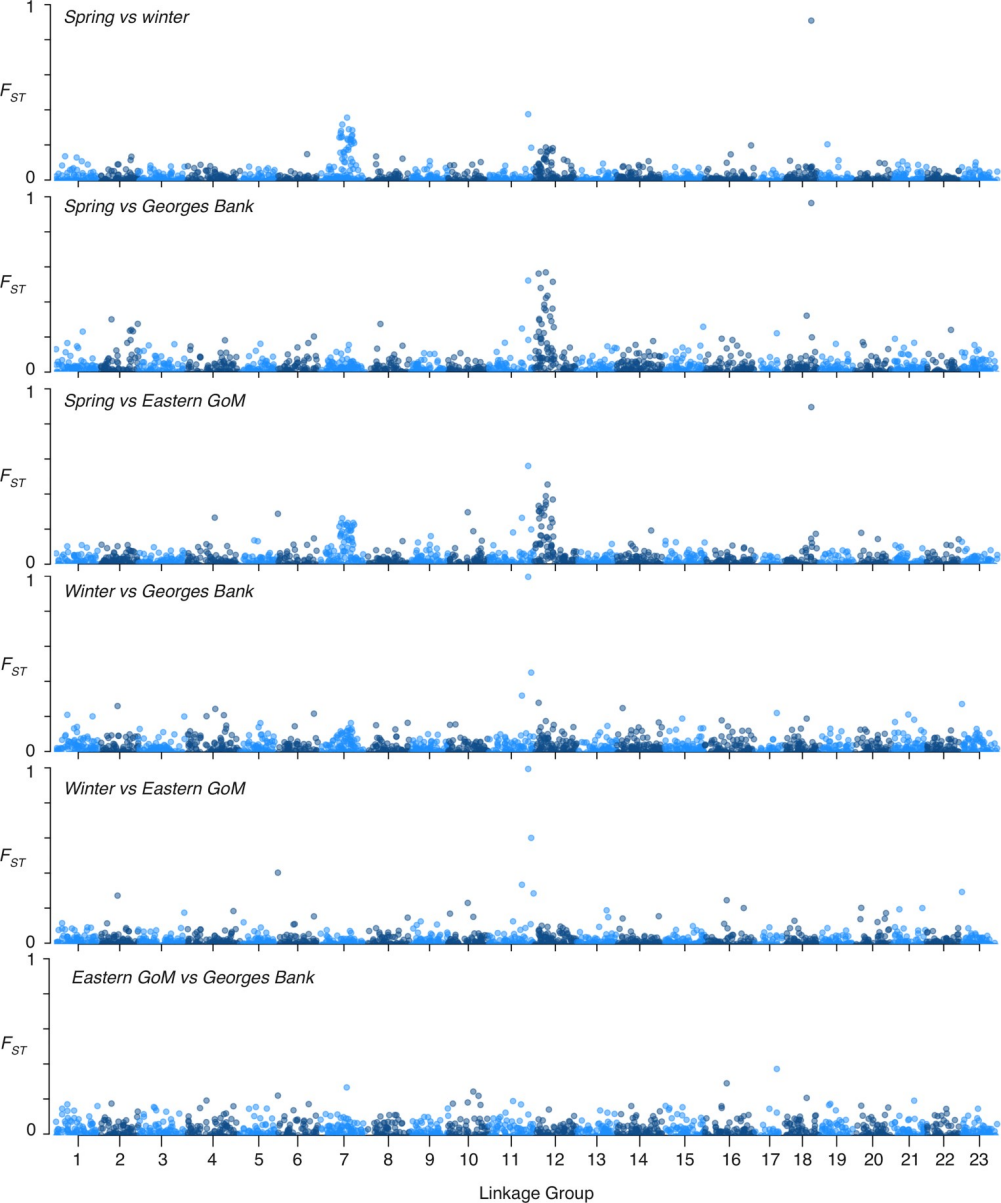
*Manhattan plots showing pairwise*, *locus-specific* F_ST_ *values between groups for the full SNP dataset*.

To investigate patterns of differentiation at the genome-wide scale, we plotted the locus-specific F_*ST*_ values for all pairwise comparisons between groups (with winter and spring spawners from Ipswich Bay and Massachusetts Bay grouped together by spawning season given the genetic similarity of these groups). Our full dataset included 62 SNPs from the inversion on LG7 and 61 from the inversion on LG12, making it possible to see the influence that these inversions were having on the inferred population structure (Fig 5). The inversion on LG 7 appeared to segregate between winter and spring spawners, between spring spawners and eastern GoM cod, and to a lesser extent between winter spawners and Georges Bank spawners. The inversion on LG 12 appeared to segregate between spring spawners and Georges Bank spawners, between spring spawners and eastern GoM cod, and to a lesser extent between winter and spring spawners. Since our dataset only included 28 SNPs from the inverted region on LG2, we were not able to make inference about the contributions of this inversion. We also found evidence for adaptive variation outside of the known chromosomal inversions: one SNP on LG 18 showed very high (> 0.8) F_*ST*_ between spring spawners and all other groups, and a small number of SNPs on LG 11 also showed elevated F_*ST*_ in all comparisons except between Georges Bank versus eastern GoM cod. Overall, comparisons between spring spawners and other groups tended to show the most elevated F_*ST*_ values, while winter spawners seemed somewhat similar to Georges Bank and eastern GoM cod, the comparison between the latter two showing the overall lowest levels of differentiation.

To further investigate genetic differentiation in each putatively inverted region on LG 2, 7, and 12, we extracted the SNPs within each LD block from our full SNP dataset using the boundaries determined by Barney *et al*., [24]. We then repeated our PCA using those subsets of SNPs.

**Fig 6:**
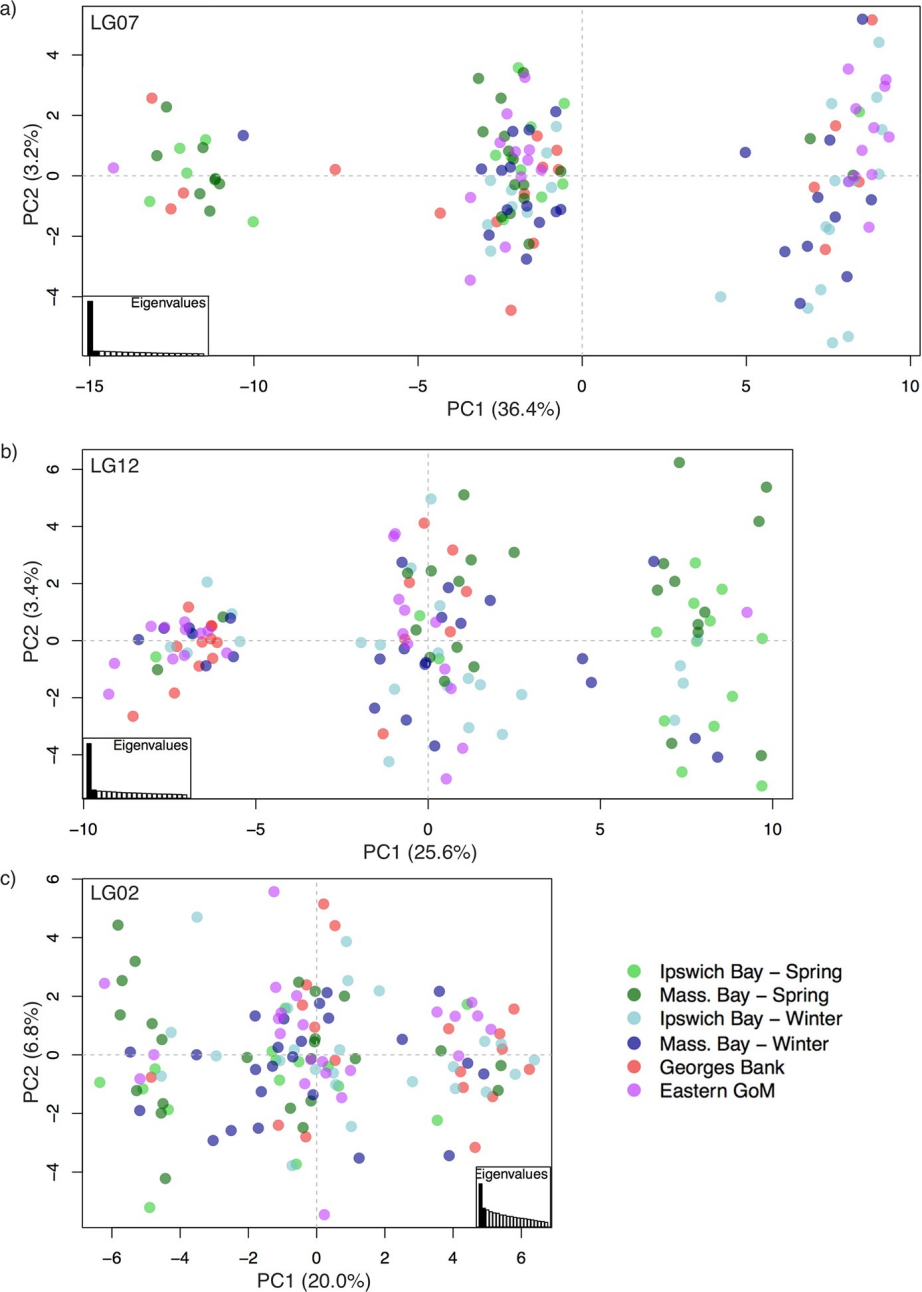
PCAs for each of the LD blocks on a) LG 7, b) LG 12, and c) LG 2. The percentage of the variance explained by each axis is shown on the axes labels.

The patterns from the inverted regions on LG 7 and 12 showed three well separated clusters in the PCAs, with PC1 explaining 36.4% and 25.6% of the variation, respectively (Fig 6A and 6B). These clusters likely corresponded to the homozygous collinear ‘genotype’, the heterozygous genotype (where individuals have one copy of the inversion and one copy of the collinear region), and the homozygous inverted genotype. ‘Genotyping’ individuals for the inversion based on which cluster they fell into showed that winter and spring spawning cod have different frequencies of the inversions. At LG 7, spring spawners were mostly homozygotes for one form of the inverted region (left-most cluster) and heterozygotes (middle cluster), while winter spawners had higher frequencies of the alternative homozygous state (right-most cluster) and many heterozygotes. Eastern GoM individuals showed a similar pattern to the winter spawners, while Georges Bank were found across all three clusters. At LG 12, spring spawners were mainly homozygous for one form of the inversion (right-most cluster) and heterozygotes (middle cluster), while winter spawners were mainly homozygous for the alternate allele or heterozygotes. Georges Bank and eastern GoM individuals showed similar haplotype frequencies as the winter spawners.

The PCA based on the 28 SNPs from the inversion on LG 2 was not as informative (Fig 6C), which is not surprising given the small number of SNPs and the overall lack of elevated F_*ST*_ values in this region (Fig 5). However, three weakly defined clusters were visible on the PCA that related to each of the homozygous states and the heterozygous state for the inversion. Groups were not well differentiated with these 28 SNPs on LG 2, with members of each group found in all three clusters, but some separation of spring spawners primarily in the left and central cluster and winter spawners primarily within the right and central clusters could be seen. Georges Bank individuals grouped primarily in the right and central clusters, similar to the winter spawners, with fewer individuals in the left cluster. No additional clustering was apparent when additional PCs were plotted for any of the LD blocks.

## Discussion

### Population structure

From our genome-wide analysis of population structure, we found evidence for differentiation among three major spawning groups: cod that spawn in the spring in Ipswich and Massachusetts Bays, cod that spawn in the winter in the same bays, and cod spawning on the northeast peak of Georges Bank. The spring spawning cod in the western GoM appeared to be the most genetically differentiated from the other spawning populations. Levels of genetic differentiation were low overall (small magnitude *F*_*ST*_) and primarily driven by loci that are putatively under selection, the majority of which were located in the genomic regions on LG 7 and 12 containing chromosomal inversions known to be divergent among other cod populations in both the Northeast and Northwest Atlantic [51–54,58,73]. Little genetic structure was apparent from neutral markers alone among these populations, but Georges Bank and the spring spawning western GoM population displayed some weak neutral differentiation. Divergence at outlier loci despite gene flow has been observed among Atlantic cod populations in other parts of its range [51]. Our findings suggest that selection is likely maintaining differentiation at adaptive loci, particularly among spring spawning cod that may experience divergent selection pressures due to their different spawning season.

The low neutral genetic differentiation suggests that there is some level of ongoing gene flow among these groups of cod, especially between the western GoM bays within the same spawning season. Small but significant levels of genetic differentiation are common in marine fishes as a result of their high dispersal potential and large effective population sizes [1,88], which can make interpretations challenging. Even low levels of neutral genetic differentiation can be biologically meaningful [89], but translating these low levels of genetic differentiation into useful estimates of demographic connectivity is not easy [4]. Evidence from individually tagged cod in the GoM supports a hypothesis of there being connectivity among the western GoM spawning locations within the same season, as individuals tagged on the primary Massachusetts Bay spring-spawning site have been recaptured or detected via acoustic telemetry in the vicinity of other spawning sites during the spawning season [90]. Spawning site fidelity is high [90–92], but cod may spawn in multiple nearby locations within a season [90].

Slightly greater neutral differentiation between winter and spring spawning groups and between the western GoM spawners and Georges Bank spawners suggest that gene flow is less prevalent among these groups. This finding is consistent with results from tagging studies, which reveal very little movement of GoM cod to areas outside of this region [36,38,90,93], supporting the view of demographic independence of cod in the GoM and Georges Bank. Movement patterns of spring and winter spawning cod in the western GoM have been studied extensively and suggest dispersal from the spawning grounds after spawning and return to the spawning grounds during distinct spawning seasons [35,90,93], consistent with genetic differentiation of these seasonal spawning groups.

Our findings agree with previous work that found significant genetic differentiation primarily driven by loci putatively under selection among spawning groups in the Gulf of Maine [23,28]. Barney *et al*., [24] localized this genetic differentiation to the inversions on LG 2, 7 and 12, although they focused their investigation on these genomic regions and did not investigate differentiation elsewhere in the genome. Contrary to the findings of Barney *et al*., [24], but consistent with Wirgin *et al*., [28] and Kovach *et al*., [23], we found that spring spawning cod are the most genetically differentiated of the spawning populations in the western Gulf of Maine and Georges Bank. This inconsistency between the studies is likely a result of small sample sizes, because Barney *et al*., [24] sampled ten individuals from each spawning group, so their estimates of the inversion frequencies in each population may not have been precise.

In addition to differences in the frequency of inversions on LG 7 and 12 among the groups studied, we found several outlier SNPs with elevated F_*ST*_ on LG 11 and 18, with the former segregating among all three primary groups (winter spawners, spring spawners, and Georges Bank), and the latter segregating primarily between the spring spawning groups and the others. Our small sample of genomic SNPs was insufficient to perform a genome-wide scan for selection because a much higher density of SNPs and an estimate of genome-wide linkage disequilibrium would be necessary [94]. Therefore, further investigations into these outlier loci should be conducted with a denser SNP dataset, which may uncover additional targets of selection elsewhere in the genome. In a similar fashion, our ability to detect differences in the frequency of the inversion on LG 2 was limited by the small number of SNPs we recovered in this region. This inversion was previously found to segregate between winter and spring spawning cod in Massachusetts Bay and to contain a number of genes that may have temperature-mediated functions [24]. Our dataset uncovered a relatively large number of SNPs within the inversions on LG 7 and 12, which showed frequency deviations at these sites. Differences in the frequencies of these inversions among populations are likely the result of divergent selection because cod are thought to have large effective population sizes [95–97] resulting in weak genetic drift.

There is a growing body of evidence from a variety of taxa that chromosomal inversions can capture multiple adaptive alleles governing complex traits and therefore act as ‘supergenes’ (e.g. *Anopheles* mosquitoes [98], three-spine sticklebacks [99], *Heliconious* butterflies [100]). In Atlantic cod, the inversions on LG 1, 2, 7, and 12 that segregate as biallelic loci between cod populations and ecotypes [49,50,54,55,57,58], may also be acting as supergenes housing suites of genes with adaptive phenotypic effects. The inversion on LG 7 has previously been linked to variation in temperature in both the Northwest and Northeast Atlantic [53], variation in salinity and oxygen concentrations in the Baltic Sea [101], and with migratory and resident ecotypes in the Northeast Atlantic that also experience different temperature regimes [57,102]. The inversion on LG 12 has also been linked to temperature [53,55,56] and with inshore and offshore populations on the Skagerrak coast of Norway that show variation in their tendency to migrate [54]. Barney *et al*., [24] identified 306 and 407 genes within the inverted regions on LG 7 and 12, respectively, but they did not find significant enrichment for any specific genomic pathways, and so how these regions could be adaptive under different temperature regimes is currently unknown.

Consistent with findings from other Atlantic cod populations, temperature and/or offshore and vertical (depth) movement patterns may also be selective pressures that maintain the polymorphisms that we observed. Spring spawning cod in the western GoM differed from the winter GoM and the Georges Bank spawners in the frequencies of the inversions on both LG 7 and 12 (and LG 2 according to REF 24), suggesting they experienced divergent selective pressures from those groups. Winter spawning cod in the western GoM appeared to be under similar selective pressures as cod spawning on Georges Bank, based on more similar frequencies of the inversions (although they are not fully connected by gene flow at neutral loci, supporting the divergence of these spawning groups). Divergent temperature-associated selection pressures could act at different life stages (e.g., egg development, larval growth, juvenile settlement, adult migration). For example, eggs that are spawned in the spring in the western GoM will experience warmer waters (based on surface temperature) than those spawned in the winter at the same location and on Georges Bank (Georges Bank cod also spawn in the winter). However, depth differences during juvenile settlement may also be a factor; spring-spawned juveniles settle at greater depths (up to 80 m) and a narrower range of temperatures (<10°C) compared with winter-spawned juveniles, which settle in shallower inshore waters (< 30 m) at a greater range of temperatures (5–15°C; ref [103]; M. Dean Massachusetts Division of Marine Fisheries, *pers*. *comm*.). Lastly, temperature differences may also be associated with adult movement patterns if populations differ in their offshore migration patterns and their affinity for deeper, cooler waters outside of the spawning season. Spring spawning cod in Massachusetts Bay move to offshore feeding grounds in the summer and fall, and then overwinter in deep (>150 m) offshore basins [90]. Further work identifying the inversion haplotypes of the different spawning aggregations and associating them with life history data is needed to fully understand the effects of temperature, depth, or migration-driven selection on these spawning populations.

### Eastern GoM

The eastern GoM cod caught by the sentinel fishery showed some of the lowest levels of neutral genetic differentiation from spawning cod from Ipswich Bay, but this differentiation increased when outlier loci were included. If we assumed that the cod caught in the eastern GoM were residents to that area, then this would suggest that there may be a high level of connectivity between Ipswich Bay and the eastern GoM, and that selection may result in the putatively adaptive allelic frequency differences that we observed, favoring the genotypes of the winter-spawned Ipswich cod. One possibility is that cod from Ipswich Bay could have moved in to fill the vacant niche after spawning aggregations largely disappeared from the eastern GoM [19]. Alternatively, connectivity may have pre-dated the loss of spawning aggregations in the eastern GoM and may represent a long-term phenomenon. However, evidence to support high connectivity between eastern and western GoM is lacking. Cod tagged in the western GoM have rarely been recaptured outside of the western GoM [35,90], supporting the idea of high spawning site fidelity to the western GoM. An alternative scenario is that the eastern GoM cod we sampled were from small, remnant spawning aggregations that have persisted in the region but have gone undetected. Differences at outlier loci on LG11 between both the winter and spring spawning western GoM cod and the eastern GoM cod point toward a separate origin of these cod, perhaps as a remnant of the historically larger aggregations.

Compared to Ipswich Bay, the eastern GoM cod showed a higher level of neutral differentiation with Georges Bank cod, suggesting there could be demographic separation of these two groups. However, putatively adaptive differentiation was low, which may suggest that similar selection pressures act on cod from eastern GoM and Georges Bank. Perhaps cod in these areas experience a similar temperature regime resulting from similar oceanographic conditions, or they may exhibit similar habitat preferences. However, we cannot confirm these hypotheses given the little information we have about these eastern GoM fish and the lack of known spawning in this region. Tagging studies conducted over the last 40 years have shown some migratory connectivity between Georges Bank, the Scotian Shelf, and the Bay of Fundy [34,35]. Few cod with conventional tags have been recaptured in eastern GoM [35], but the few recaptures likely reflect the relatively low intensity of fishing in the eastern GoM. Fishery-independent inferences of cod movement from geolocation of archival tags suggest relatively high connectivity between western and eastern Gulf of Maine is possible during certain seasons [90]. Therefore, cod caught by the sentinel fishery in the eastern GoM could have been recent migrants from adjacent areas.

Our results do not provide a clear conclusion to the source of the cod caught by the sentinel fishery in the eastern GoM, because signals of neutral and adaptive genetic variation do not align to consistently identify them with either the western GoM or Georges Bank spawning populations. It is also possible that the cod caught in this area today are a mixed stock, resulting from foraging migrations of cod that spawn in other areas. Indeed, the samples we analyzed in this study were collected during a 3-month period (June–August), during which cod could have moved into this area from different locations (including possibly a mix of Canadian and U.S. waters). Further studies using genetic data from historical samples (e.g., otolith collections) pre-dating the collapse of the eastern GoM stocks are needed to definitively test the hypothesis about demographic separation of this population in the past. These studies could indicate whether there has been long-term connectivity between eastern GoM and other areas, which would be informative for investigating potential mechanisms for rebuilding of cod in the eastern GoM.

### Implications for stock structure

The patterns of fine-scale population structure that we found have been confirmed by several studies [23,24,28] and are in contrast to the current two stock strategy for assessment and management. This strategy groups all eastern and western GoM cod into one stock, and cod from southern New England to Georges Bank into a second, southern stock (Fig 1). After conducting an interdisciplinary review, Zemeckis *et al*., [20] suggested that there may be five separate subpopulations of cod in US waters. These subpopulations correspond to: 1) an eastern Georges Bank population that spawns in the winter and early spring; 2) a northern spring coastal complex that spawns in the spring in Ipswich Bay, Massachusetts Bay, and Bigelow Bight; 3) a southern complex that includes winter spawners from Ipswich Bay, Massachusetts Bay, and Jeffreys Ledge, early-spring spawners from Stellwagan Bank, and winter spawners from Nantucket Shoals, the Great South Channel, southern New England and the Middle Atlantic; 4) a mid-coast GoM population; and 5) an eastern GoM population. The latter two subpopulations were defined by Ames [19] and are now severely depleted, with no spawning observed in these areas for the last two decades. Our results provide further evidence consistent with the metapopulation model proposed by Zemeckis *et al*., [20] and show that the current GoM stock encompasses multiple, genetically distinct sub-populations that likely harbor different local adaptations. The lack of recognition in the current management system of the two seasonally distinct western GoM subpopulations and a third possibly distinct subpopulation in eastern GoM may put this biocomplexity at risk.

Despite high dispersal potential in marine species at either larval or adult stages, local adaptation is common (e.g., purple sea urchin, *Strongylocentrotus purpuratus* [104], grey reef sharks, *Carcharhinus amblyrhynchos* [105], hamlet fish, *Hypoplectrus* spp., [106]) and local adaptation has often been linked to temperature (e.g. American lobster, *Homarus americanus*, [107], sea scallops, *Placopecten magellanicus*, [108], northern shrimp, *Pandalus borealis*, [109]). Conserving locally adapted populations should be a high priority for fishery managers, because the functional aspect of adaptive genetic diversity is arguably more important to preserve than neutral genetic diversity [8]. Adaptive genetic diversity is also one of the components that determines the adaptive capacity of species and populations [12], because species with higher genetic diversity (both neutral and adaptive) have a greater probability of surviving rapid environmental change through adaptation. Atlantic cod are likely to be severely affected by the rapid warming of the Gulf of Maine, which has warmed faster than 99% of the global ocean [44]. Therefore, preserving adaptive genetic diversity, especially if it is linked to thermal regime, is likely going to be critical for this species into the future.

Preserving population diversity or biocomplexity is also critical for fishery stability and resilience by providing diverse responses to the environment [5,15,16]. Portfolio effects, whereby a high diversity of discrete populations reduces the variability in a stock [5,11], can provide stability and resilience in fisheries [15]. The loss of Atlantic cod spawning components [19] has likely impacted the productivity and stability of the population and the fishery targeting this resource. Furthermore, the adaptive differences among spawning populations suggest that the potential for severely depleted populations to recover through migration from other spawning populations could be limited, because immigrants may have low fitness due to lack of suitable local adaptations [110]. Accordingly, preservation of the remaining biocomplexity of Atlantic cod within the Gulf of Maine and Georges Bank should be considered in future fishery management efforts.

## Supporting information

S1 Appendix Supplementary information. Contains supplementary table A and supplementary figures A—H.
(PDF)
