# Supplementary material for: Adaptive genetic variation underlies biocomplexity of Atlantic Cod in the Gulf of Maine and on Georges Bank
Source: PLoS One. 2019 May 24;14(5):e0216992. doi: 10.1371/journal.pone.0216992 (PMC6534298; doi:10.1371/journal.pone.0216992)
Supplement: S1 Appendix — Contains supplementary table A and supplementary figures A—H. (PDF) [file pone.0216992.s001.pdf]

**SUPPLEMENTARY INFORMATION**

# Adaptive Genetic Variation Underlies Biocomplexity of Atlantic Cod in the Gulf of Maine and on Georges Bank

G.V. Clucas, L.A. Kerr, S.X. Cadrin, D.R. Zemeckis, G.D. Sherwood, D. Goethel, Z. Whitener, and A.I. Kovach.

*Supplementary Table A. The number of SNPs retained after each filter was applied to our initial dataset of 73,495 SNPs.*

| Filter | Number of SNPs retained after applying each filter |
|---|---|
| No filter | 73,495 |
| Biallelic SNPs only | 67,264 |
| MAF > 0.05 | 9,188 |
| Single SNP per RADtag | 5,756 |
| < 30% missing data per sampling location | 3,250 |
| HWE in at least 3 sampling locations | 3,225 |
| Depth within 2 SD of mean | 3,128 (full dataset) |
| At least 10 kb apart | 2,834 |
| $F_{ST}$ outliers removed | 2,795 |
| SNPs in LD blocks on LG 02, 07 and 12 removed | 2,689 (neutral dataset) |
| Full dataset but with < 5% missing data across all sampling locations | 1,660 (stringent dataset) |

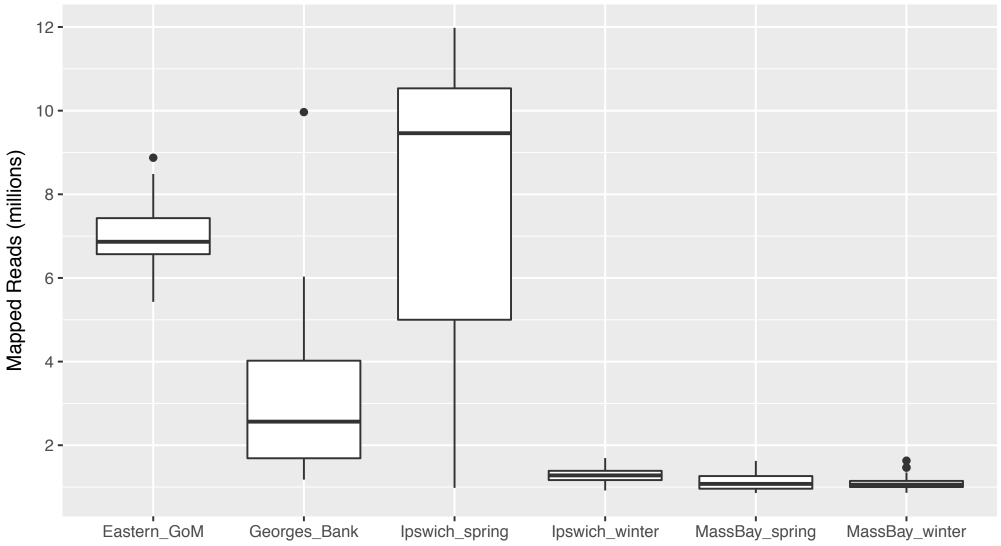


*Supplementary Figure A. Boxplots of the number of mapped reads per individual in each sampling location.*

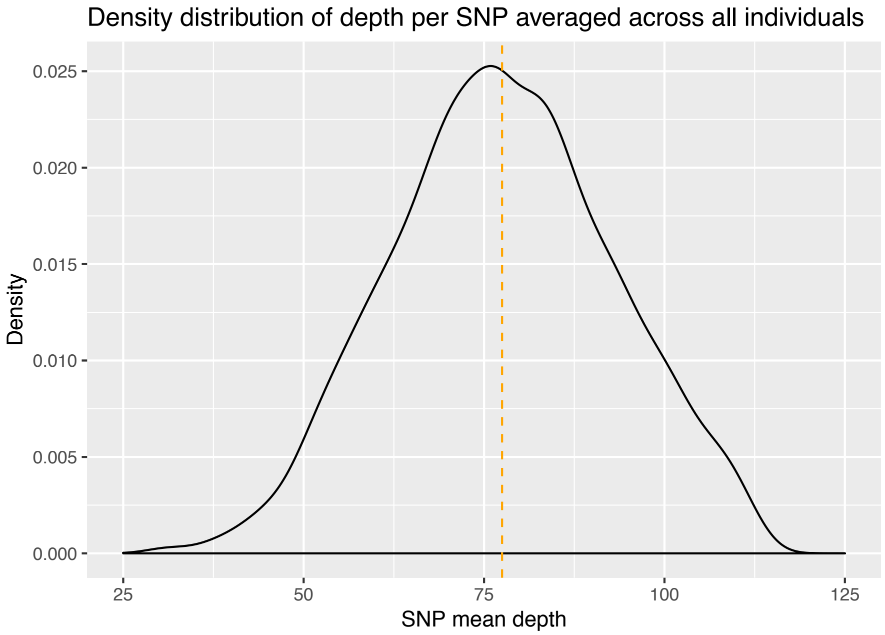


*Supplementary Figure B. Density distribution of the mean depth per SNP in the full SNP dataset when averaging across all individuals. The orange dotted line marks the mean of the distribution.*

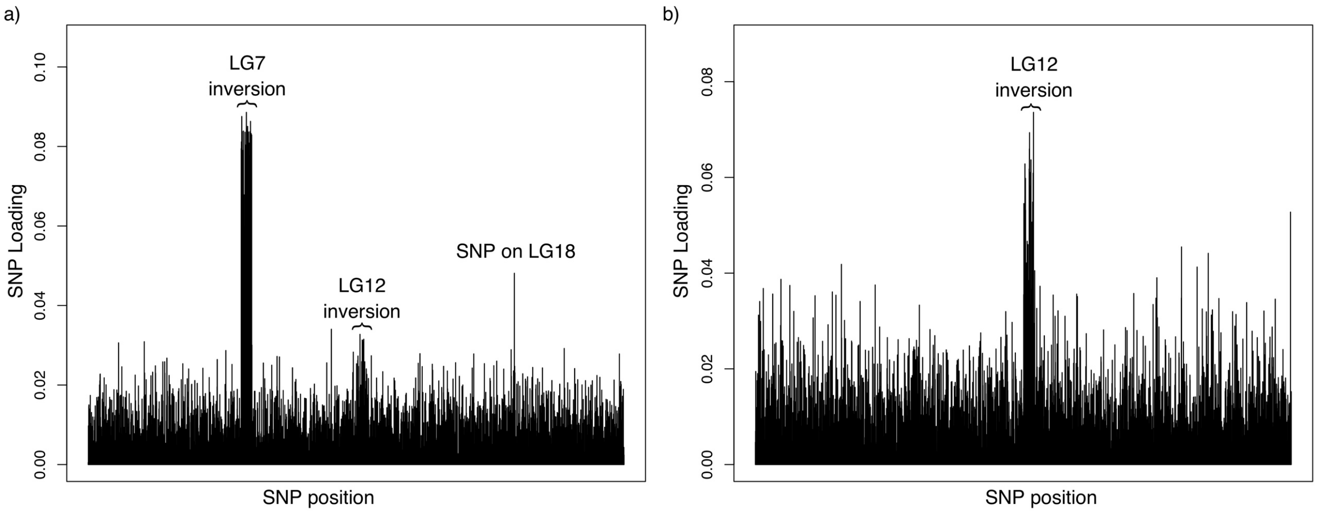


*Supplementary Figure C. Loading plot showing the individual allele contributions to a) PC1 and b) PC2.*

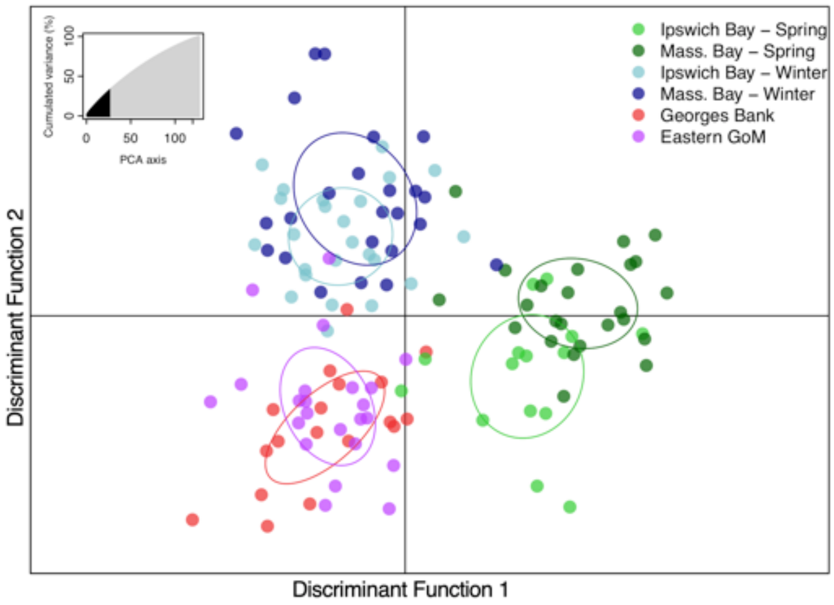


*Supplementary Figure D. DAPC using the stringent SNP dataset of 1660 SNPs generated by allowing only 5% missing data. The cumulative percentage of variation explained by the discriminant functions is shown on the inset graph. The number of principle components retained was 27, as assessed using cross-validation. The structure recovered in this stringent analysis was identical to that recovered by the full dataset.*

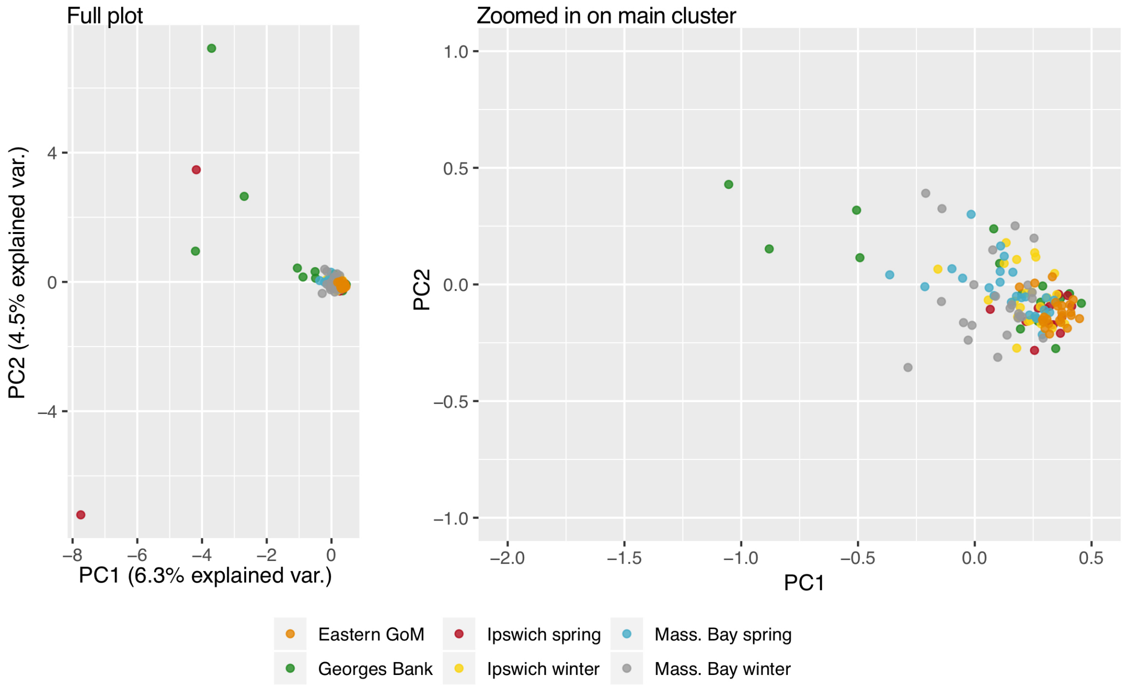


*Supplementary Figure E. A PCA to investigate the effect of missing data on population structure. Points represent individuals, which are coloured according to their sampling location. The left-hand panel shows the full PCA plot and the amount of variance explained by the PCs is displayed on the axes labels. The right-hand plot is zoomed in to the main cluster of points. The scattering of points confirms that there is no pattern of population structure in the missing data.*

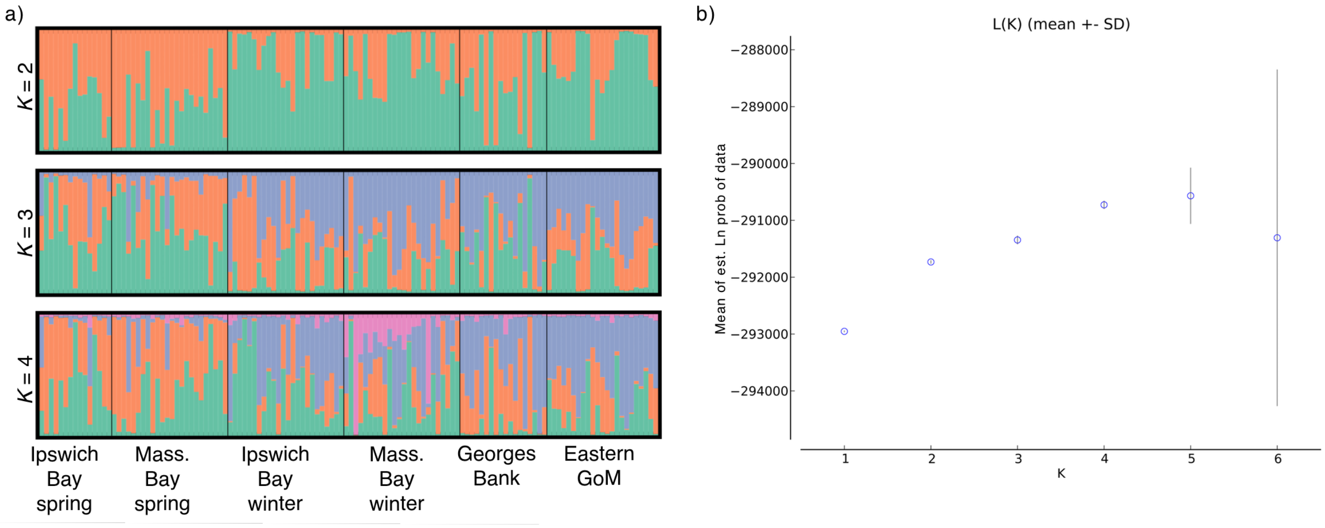


*Supplementary Figure F. Structure results from ten replicate runs of Structure using the full SNP dataset without location priors. A) Estimated ancestry proportions for each individual to each cluster when the number of clusters (K) is varied from two to four; b) the mean estimated posterior probability of the data when K was varied from one to six.*

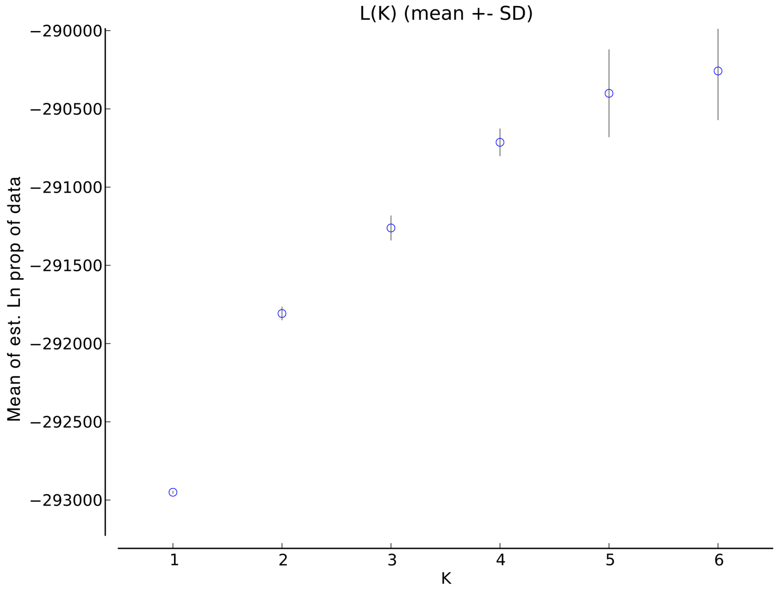


*Supplementary Figure G. The mean estimated posterior probability of the data for K one to six for the full SNP dataset with location priors.*

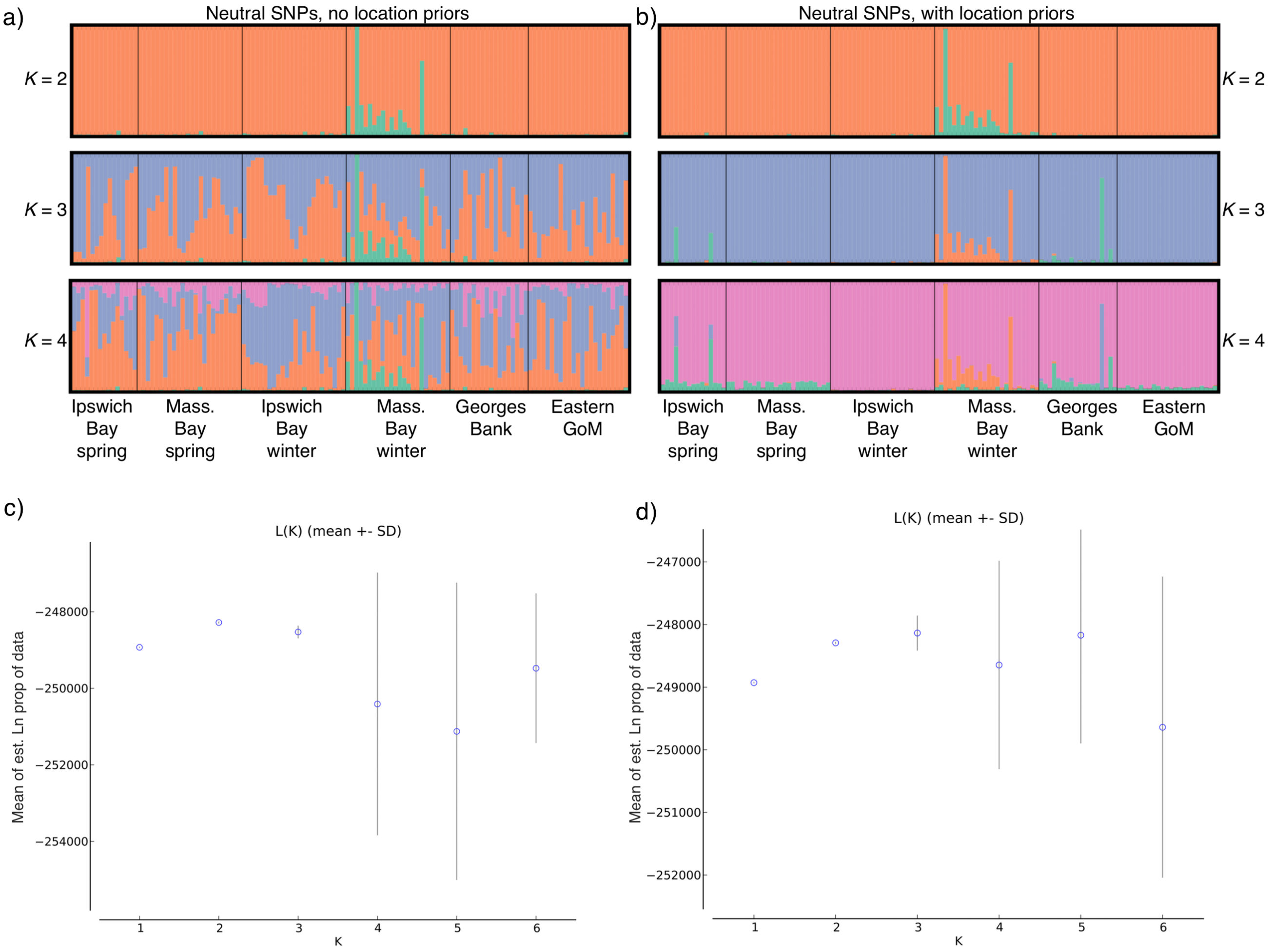


*Supplementary Figure H. Structure results aggregated from ten replicate runs of Structure using the neutral SNP dataset, with and without location priors. Estimated ancestry proportions for each individual to each cluster when K is varied from two to four are show without location priors in (a) and with location priors in (b). The signal in Mass Bay winter at K = 2 is largely driven by two outliers in the dataset. The mean posterior probabilities of the data are shown for K = 1 to K = 6 with location priors in (c) and without location priors in (d).*
